# Capreomycin-induced optic neuritis in a case of multidrug resistant pulmonary tuberculosis

**DOI:** 10.4103/0253-7613.68436

**Published:** 2010-08

**Authors:** Rahul Magazine, Mahuya Pal, Bharti Chogtu, Veena Nayak

**Affiliations:** Department of Pulmonary Medicine, Kasturba Medical College, Manipal University, Manipal, Karnataka, India; 1 Department of Ophthalmology, Sikkim Manipal Institute of Medical Sciences, Gangtok, Sikkim, India; 2 Department of Pharmacology, Kasturba Medical College, Manipal University, Manipal, Karnataka, India

**Keywords:** Capreomycin, optic neuritis, tuberculosis

## Abstract

A patient of multidrug-resistant pulmonary tuberculosis was prescribed an anti-tubercular regimen containing capreomycin. Patient developed optic neuritis 3 months after starting treatment. Investigations did not reveal any specific cause for this ocular condition and on discontinuing capreomycin his vision recovered. We conclude that capreomycin is the cause of reversible optic neuritis in our case.

## Introduction

Optic neuritis is an inflammation of the optic nerve resulting in painful loss of vision. The etiology includes idiopathic optic neuritis, multiple sclerosis, infections, and autoimmune diseases.[1] Optic neuritis has also been reported with anti-tubercular drugs like ethambutol, isoniazid, and rarely streptomycin.[2] Capreomycin is a second line anti-tubercular drug used in the treatment of multidrug-resistant tuberculosis. The chemical structure of capreomycin is different from that of aminoglycosides, but the mechanism of action is similar. However, it does not show cross-resistance with the aminoglycosides.[3] To the best of our knowledge capreomycin-induced optic neuritis has not been reported in the literature. We present a case of optic neuritis following use of capreomycin in a patient of multidrug-resistant pulmonary tuberculosis.

## Case Report

A 26-year-old unmarried male presented to the chest OPD with history of cough, expectoration and low grade fever of 1 year and 6 months duration. General physical examination was normal. Chest examination revealed coarse crepitations in the right infraclavicular area. Examination of other systems revealed no abnormality. He had defaulted on first line anti-tubercular regimen. Based on sputum culture and sensitivity report, patient was managed as a case of multidrug-resistant pulmonary tuberculosis. He was prescribed levofloxacin, ethionamide, pyrazinamide, clarithromycin, and clofazimine for last 1 year. During the first 2 months of treatment he was also given injection kanamycin.

Despite taking second line regimen for 1 year, his sputum smear was positive for acid fast bacilli. The sputum culture and sensitivity grew bacilli of *Mycobacterium tuberculosis* which were resistant to all first line drugs, *para*-aminosalicylic acid and ofloxacin. He was treated with capreomycin, cycloserine, amoxicillin/clavulanate, linezolid, pyrazinamide, and pyridoxine at the WHO recommended dosage schedules. The patient was reviewed weekly to screen for any drug-related side effects. The patient was HIV negative; hemogram, blood biochemistry, and serum electrolytes were within normal limits at the start of this regimen. The Mantoux test showed 11 mm× 10 mm induration. After 3 months he complained of diminution of vision and pain in both the eyes. Ophthalmologic examination revealed a marked diminution of vision in both eyes to finger counting at 3 m and deficient color vision. Visual field estimation could not be performed due to decreased vision. Anterior chamber examination was normal except that the pupils were sluggishly reacting to light. Fundoscopic examination by indirect ophthalmoscopy and by +90 Diopter lens in the slit lamp biomicroscopy revealed significant pallor of the disc in both the eyes. On retinoscopy no uncorrected refractive error was detected, suggesting optic neuritis. MRI of brain and work up for connective tissue disorders did not show any specific cause for optic neuritis. Linezolid was suspected to be the offending agent and was discontinued. In view of the seriousness of his chest condition other drugs were continued. However, in spite of discontinuing linezolid for 2 weeks the visual loss worsened. As optic neuritis has been reported to occur rarely with injectable anti-tubercular drug such as streptomycin[4] we withdrew capreomycin from the regimen. There was a significant improvement in his vision over next 2 weeks and normalization after another 8 weeks. After 4 weeks of stopping capreomycin, linezolid was reintroduced and continued for another 2 months. After a total of 24 months of therapy the patient was declared cured based on clinical, radiological, and microbiologic data. During this period he did not report any major side effect or visual disturbances.

## Discussion

We attribute optic neuritis to capreomycin in our case because even 2 weeks after stopping the linezolid there was deterioration in the vision and it was only after withdrawing capreomycin that visual improvement started. The causality relationship was established. We conclude that the reaction can be put in the category of probable/likely adverse drug reaction with capreomycin.[5] As per Naranjo algorithm the causality assessment score was 5 which also categorizes this as a probable adverse drug reaction.[6] Our patient had taken most of the second line anti-tubercular drugs except capreomycin and cycloserine. For prescribing an adequate regimen four to five drugs are needed, we used drugs belonging to WHO group 5 like linezolid and amoxicillin/clavulanate. The dosage of capreomycin used was 15 mg/kg/day.[3] In one of the case reports, linezolid has been reported to cause optic neuritis which improved 2 weeks after discontinuing it and then returned to normal in 3 months.[7] However, in our case, the vision deteriorated even after stopping linezolid. Even though capreomycin is a cyclic polypeptide, its side effect profile is similar to that of aminoglycosides. This prompted us to consider it as the cause of optic neuritis and decide to withdraw it from the regimen. After discontinuing capreomycin the vision improved and the patient recovered completely over next 8 weeks. This suggested that capreomycin was the probable cause of diminished vision in this case. Incidence of visual disturbances and blurred vision have been reported with gentamicin as well.[8]
